# Robotic surgery for rectal cancer in 2026: a hierarchical appraisal of the evidence

**DOI:** 10.1007/s11701-026-03671-4

**Published:** 2026-07-20

**Authors:** Pedro Silva-Vaz, Mariana Belo Cruz, José Guilherme Tralhão

**Affiliations:** 1 General Surgery Department, Coimbra University Hospitals, Unidade Local de Saúde de Coimbra, Coimbra, 3000-075 Portugal; 2 Faculty of Medicine, University of Coimbra, Coimbra, 3000-548 Portugal; 3 Coimbra Institute for Clinical and Biomedical Research (iCBR) Area of Environment Genetics and Oncobiology (CIMAGO), Faculty of Medicine, University of Coimbra, Coimbra, 3000-548 Portugal; 4 CNC.IBILI Consortium/Center for Innovation Biomedicine and Biotechnology (CIBB), University of Coimbra, Coimbra, 3000-548 Portugal; 5 Clinical Academic Center of Coimbra (CACC), Coimbra, 3000-561 Portugal

**Keywords:** Robotic Surgery, Rectal Cancer, Total Mesorectal Excision, Standard of Care, Cost-effectiveness, Functional outcomes

## Abstract

Robotic total mesorectal excision (TME) now rests on an evidence base that has accumulated layer by layer: randomized trials, meta-analyses, registry cohorts, and dedicated functional-outcomes studies. The publication of the long-term results of the REAL trial (2025) marked a turning point. We propose a hierarchical evidence framework in which the REAL trial functions as confirmatory apex rather than foundational basis. It provided the first randomized evidence of better locoregional control and disease-free survival with robotic surgery compared to laparoscopy (3-year locoregional recurrence 1.6% vs. 4.0%; absolute risk reduction 2.4%; HR 0.45). This narrative review synthesizes the current evidence comparing robotic TME with laparoscopic TME, framing the most recent randomized data within the broader context of the accumulated literature. Across multiple study designs, robotic TME is consistently associated with lower conversion rates, better circumferential resection margin negativity in selected subgroups, and better preservation of urinary and sexual function. These advantages are amplified in male patients, those with obesity, post-neoadjuvant fibrosis, and lower rectal tumors. Important caveats remain: REAL was conducted exclusively at high-volume Chinese centers, an overall survival benefit has not yet been demonstrated, and cost and access barriers continue to shape global adoption. Evidence in 2026 supports robotic TME as the preferred minimally invasive approach for mid-to-low rectal cancer in centers with established expertise, adequate infrastructure, and case volume. When these conditions are not met, expert laparoscopic TME retains its place. Five-year data and validation across diverse healthcare systems are needed before a universal recommendation can be made.

## Introduction

Colorectal cancer remains one of the most prevalent malignancies worldwide, with rectal cancer accounting for approximately one-third of all colorectal cancer diagnoses [1]. In Europe, an estimated 130,000 new cases of rectal cancer are diagnosed annually; in Portugal, national registry data indicate 1,200 new rectal cancer cases per year [2]. Despite improvements in multimodal therapy, including neoadjuvant chemoradiotherapy, total neoadjuvant therapy (TNT), and targeted biological agents, surgical resection with curative intent remains the cornerstone of treatment for non-metastatic rectal cancer [3, 4].

Over the past century, the surgical management of rectal cancer has undergone a transformation without parallel in oncological history [5, 6]. Once regarded as a uniformly fatal disease, the convergence of anatomical understanding, surgical technique, and multimodal therapy has reduced operative mortality to under 4% in high-volume centers, a trajectory that mirrors, and in many ways defines, the broader evolution of surgical oncology [7–9]. The introduction of total mesorectal excision (TME) by Heald and colleagues in the 1980s reduced local recurrence rates from over 30% to below 10% in specialized centers [10]. The transition from open to laparoscopic TME, validated through the COLOR II, COREAN, ACOSOG Z6051, and MRC CLASICC randomized trials in the 2010s, established minimally invasive surgery as the standard approach based on equivalent oncological outcomes and consistent short-term advantages [11–17]. Table 1 summarizes the principal milestones of this evolution.

**Table 1:**
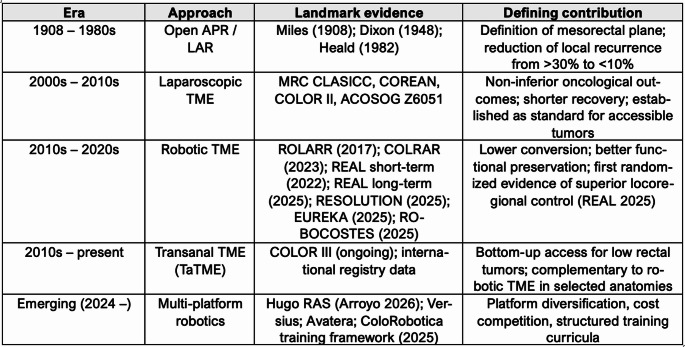
Evolution of surgical approaches in rectal cancer – landmark trials and conceptual milestones

*APR* = abdominoperineal resection; *LAR* = low anterior resection; *TME* = total mesorectal excision; *TaTME* = transanal total mesorectal excision

However, laparoscopic TME has significant technical limitations, particularly in the anatomically constrained male pelvis, obese patients, and following neoadjuvant radiotherapy [18]. The two-dimensional optical field, rigid instrumentation, restricted range of motion, and awkward ergonomics in deep pelvic dissection collectively impose a steep learning curve and an inherent ceiling on surgical precision [19]. These limitations have provided the impetus for the development and adoption of robotic-assisted surgery.

Robot-assisted TME has followed a different trajectory. Robotic surgery has been evaluated across a growing body of evidence addressing conversion rates, pathological quality, pelvic nerve preservation and, most recently, locoregional recurrence [20–22]. The reported differences appear most apparent in the anatomically demanding environment of the mid and low pelvis, where the limitations of rigid laparoscopic instruments are most consequential. The robotic platform’s degrees of freedom, motion scaling, tremor filtration, and stereoscopic three-dimensional visualisation provide capabilities that conventional minimally invasive surgery cannot replicate in this constrained operative environment, enabling correct triangulation, traction, and counter-traction at depth, and facilitating more precise dissection adjacent to the autonomic nerve structures of the pelvic floor [23, 24].

The functional dimension of this argument is equally compelling; preservation of urinary, sexual, and anorectal function is a major determinant of long-term quality of life. The systematic advantages in autonomic nerve preservation demonstrated by robotic dissection, translating into lower rates of urinary retention and superior sexual function recovery in both males and females, represent a quality-of-care imperative that extends beyond the oncological argument and is increasingly supported by prospective functional outcome data [25–27].

The economic argument, historically a substantial objection to robotic adoption, has evolved. The traditional position, higher capital and per-case costs without proportional clinical benefit, has been challenged by prospective cost-effectiveness data from the ROBOCOSTES trial, which reported favorable cost-utility outcomes for robotic rectal resection compared with laparoscopic in the Spanish healthcare context, driven by reduced blood loss, postoperative pain, and hospital readmission rates [28]. Institutional analyses further corroborate that increased adoption of robotics need not raise total hospital costs when savings from fewer conversions, reduced complications, and shorter rehabilitation are incorporated [29]. Robotic costs encompass not only platform acquisition but also maintenance contracts, instrument consumables, and service agreements, and vary substantially between countries and health systems. Second-generation systems (Hugo RAS, Versius, Avatera) are entering clinical use, although their impact on the cost landscape remains to be determined [30, 31].

**Fig. 1:**
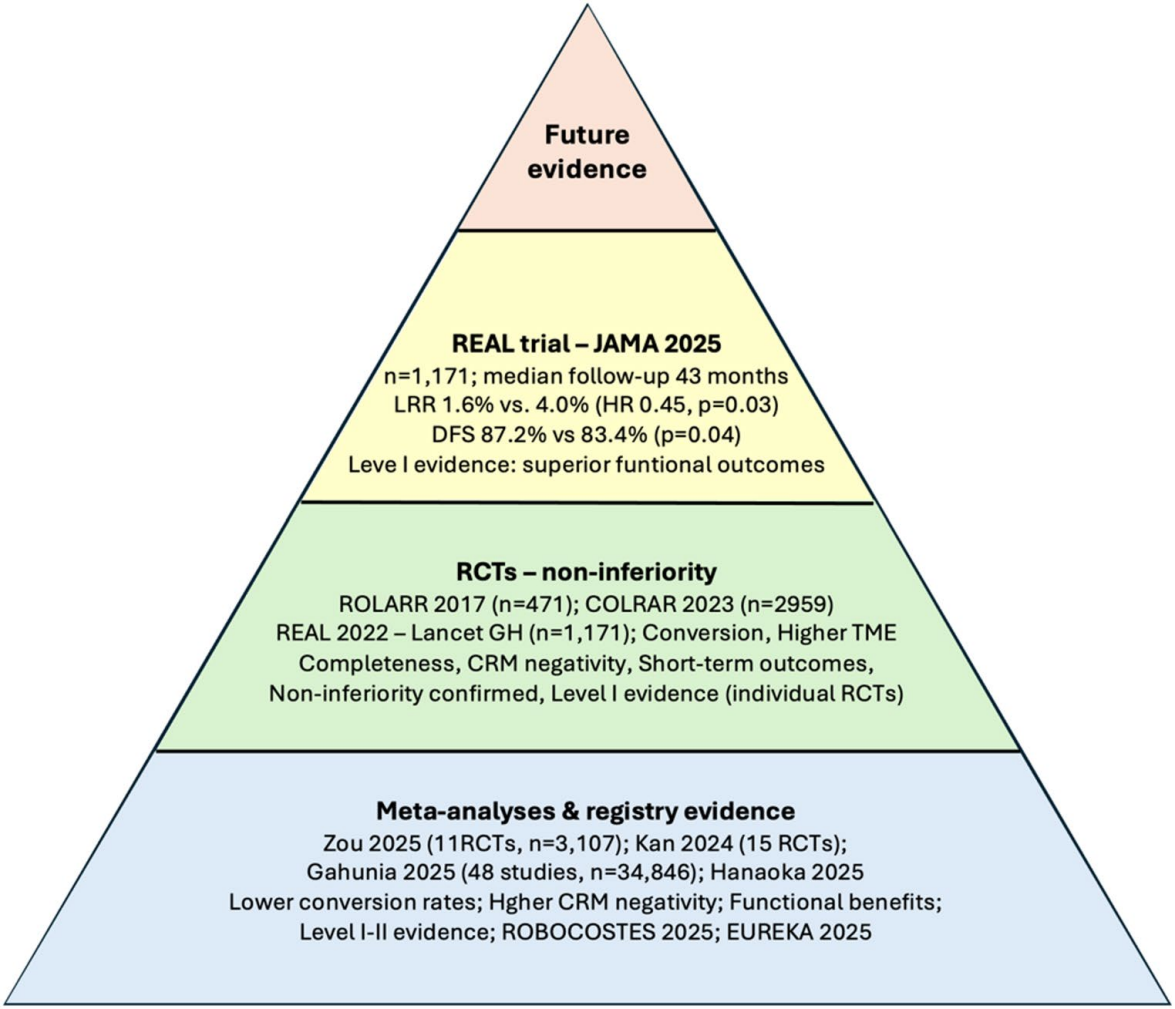
Hierarchical evidence pyramid for robotic total mesorectal excision (R-TME) in rectal cancer (2026). The base encompasses meta-analytic and registry data confirming consistent advantages in conversion rate, circumferential resection margin negativity, and functional preservation across tens of thousands of patients. The intermediate tier comprises individual randomized controlled trials establishing non-inferiority and safety. The apex represents the REAL trial (JAMA, 2025), which provided the first level I evidence of superior locoregional control and disease-free survival, confirming a hypothesis built on the layers below, not generating it *de novo. CRM = *circumferential resection margins; DFS = disease-free survival;* LRR = *locoregional recurrence rate;* RCT = *randomized controlled trial;* R-TME = *robotic total mesorectal excision

This review analyzes the evidence in the literature, evaluates the arguments in favor of designating robotic surgery as the standard treatment, and addresses the challenges that qualify universal adoption of the robotic approach in rectal surgery. The approach adopted is deliberately hierarchical: the argument in favor of robotic TME is built upon the broad base of meta-analytic and registry evidence, with the most recent randomized clinical trials serving as confirmation rather than the sole basis (Fig. 1).

## Methods

This work is presented as a structured narrative review. A narrative format was selected because the question “Is robotic surgery the standard of care for rectal cancer in 2026?” requires the integration of heterogeneous evidence: randomized controlled trials, meta-analyses, systematic reviews, and large observational cohort studies, cost-effectiveness analyses, and learning-curve literature. Additional relevant studies were identified through manual reference screening. A literature search was performed using PubMed, Embase, and Cochrane Library databases for studies published up to January 2026. Keywords included “robotic rectal surgery”, “robotic total mesorectal excision”, “laparoscopic TME”, and “rectal cancer outcomes.

The evidence was appraised using a hierarchical framework, integrating findings from meta-analyses and registry data with randomised trial evidence to provide a comprehensive evaluation of the current role of robotic TME.

## A layered appraisal of evidence in 2026

### The foundation: meta-analytic and registry evidence

The most robust starting point for evaluating robotic TME is the aggregated evidence from multiple randomized trials. One randomized controlled trial (*n* = 130) showed that robot-assisted radical resection for mid-low rectal cancer achieved short and long-term outcomes comparable to laparoscopic surgery, with the only significant advantage being reduced intraoperative blood loss [32]. Another randomised controlled trial, phase II (*n* = 139) showed that robot-assisted surgery for rectal cancer achieved TME quality, resection margins, morbidity, and bowel function recovery comparable to laparoscopic surgery, with better sexual function recovery at 12 months postoperatively in the robotic group [33]. A meta-analysis of 11 RCTs encompassing 3,107 patients confirmed that robotic surgery achieves significantly lower conversion rates (OR 0.42, 95% CI 0.28–0.63, *p* < 0.0001), lower CRM positivity, greater lymph node harvest, faster return of bowel function, and reduced reoperation rates (OR 0.454, 95% CI 0.31–0.97, *p* = 0.03) compared to laparoscopy [34]. A more recent meta-analysis of 15 RCTs reported significantly lower conversion to open surgery with robotic-assisted rectal surgery (RR 0.53, 95% CI 0.38–0.74, *p* = 0.0002), without statistically significant differences in anastomotic leak, postoperative ileus, urinary retention, surgical-site infection, or intra-abdominal abscess [35].

The advantage is not uniform across patient populations; it is amplified precisely where it matters most. A systematic review of 48 studies encompassing 34,846 patients demonstrated that robotic surgery was associated with significantly lower conversion rates in obese patients (OR 0.41), male patients (OR 0.28), and patients with low rectal tumors (OR 0.10) [36]. These subgroups represent those for whom pelvic anatomical challenges are most consequential and where laparoscopic conversion carries the greatest perioperative and oncological penalty.

Long-term observational data further reinforce these findings. The EUREKA collaborative, an international multicenter retrospective cohort across experienced Dutch, French, and United Kingdom centers (*n* = 1,390), demonstrated that robot-assisted TME performed by experienced surgeons in high-volume centers is safe and oncologically effective, with a 3.7% conversion rate, 94.7% R0 resections, 90.1% 3-year overall survival, and a 2.9% local recurrence rate [37]. A Japanese real-world registry cohort reported improved 5-year survival with robot-assisted resection compared with laparoscopic and open surgery in locally advanced rectal cancer [38]. The recently published RESOLUTION 10-year comparative cohort similarly confirmed sustained perioperative and short-term oncological advantages with robotic TME [20]. The international multicenter ALRITE study (*n* = 1,039 R-TME patients across six European countries; 2013–2020) reported a 3-year locoregional recurrence rate of 3.8%, identifying advanced clinical M-staging and postoperative complications. Pathological N-staging, completeness of resection, and resection margin distance as significant predictors; an eXtreme Gradient Boosting model achieved 77.1% accuracy and an AUC of 0.76 for recurrence prediction, further supporting the oncological safety of R-TME in high-volume centers [39]. The robotic advantage is not confined to low rectal resections; a contemporary propensity-score matched cohort of 130 patients undergoing elective anterior resection at a center with established robotic assistance was associated with significantly lower intra-operative blood loss, shorter operative duration, faster recovery of gastrointestinal function, earlier discharge, and a lower complication rate, without differences in tumor characteristics or short-term oncological outcomes [40]. This evidence is particularly relevant because earlier comparisons of robotic versus laparoscopic high anterior resections, largely based on unmatched retrospective series and earlier-generation robotic systems, had reported similar clinical outcomes at the expense of higher cost and longer operative time, suggesting that benefit was confined to low rectal resections; contemporary data from mature robotic programmes challenge that view and indicate clinically meaningful gains for both the patient and the healthcare system across the spectrum of anterior resections. Taken together, the convergence of evidence across different healthcare systems, patient populations, and study methodologies constitutes a strong evidential foundation that is independent of any single trial (Table 2).

**Table 2:**
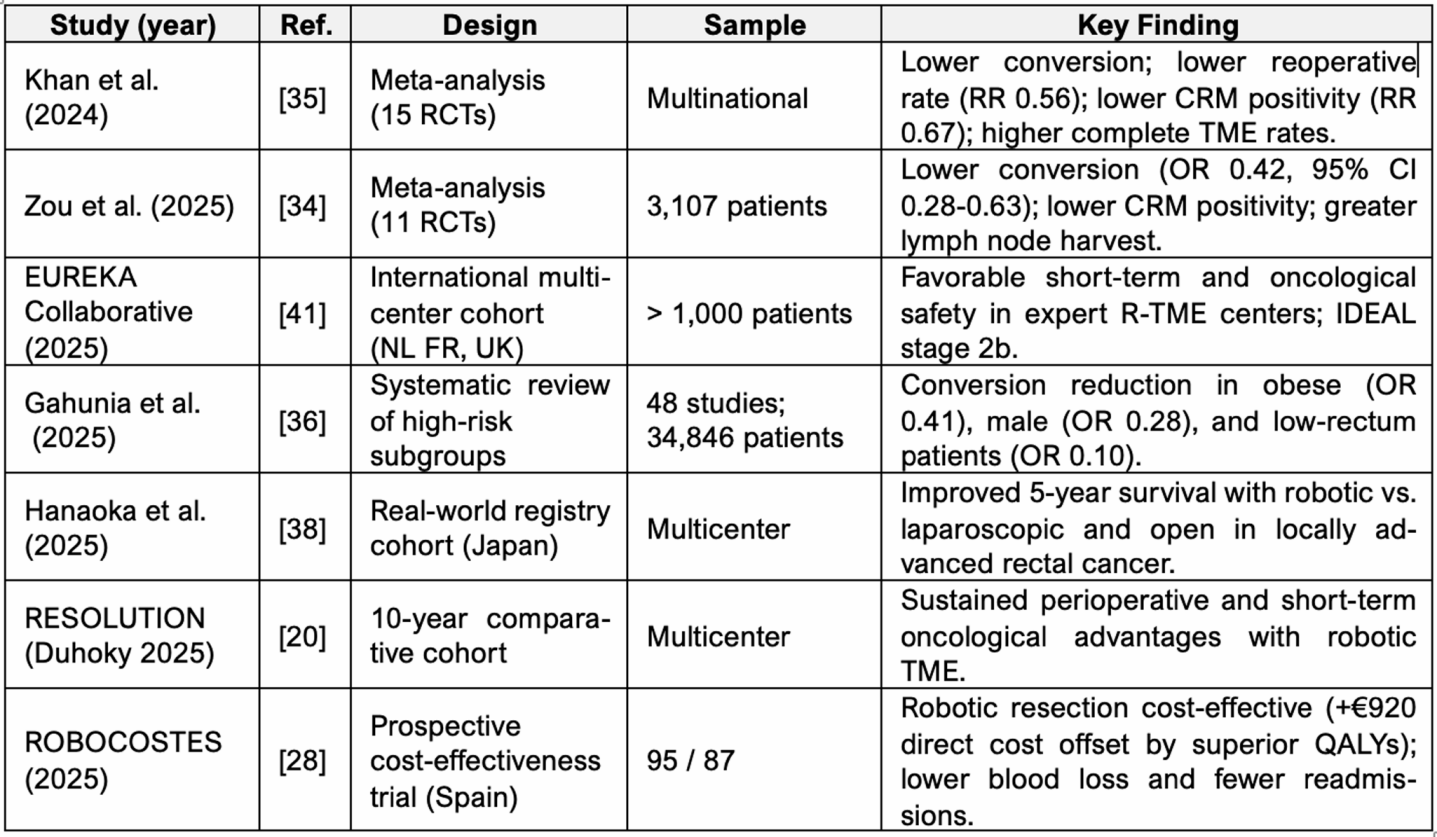
Meta-analyses, registry studies, and prospective cohorts of robotic versus laparoscopic TME (selected)

*R-TME = *robotic total mesorectal excision;* QALY = *quality-adjusted life-year;* OR = *odds ratio;* RR = *risk ratio;* CI = *confidence interval

### Functional outcomes: evidence beyond oncology

A dimension of the argument for robotic TME that meta-analyses of surgical outcomes capture incompletely is the higher functional preservation. A dedicated meta-analysis of urogenital function after robotic versus laparoscopic rectal cancer surgery demonstrated lower rates of urinary retention and superior sexual function recovery in both males and females after robotic dissection, attributable to more precise identification and preservation of the pelvic autonomic plexus [26].

These findings reflect the biological plausibility of robotic advantage: the platform’s seven degrees of freedom, motion scaling, and tremor filtration facilitate more precise dissection adjacent to the neurovascular structures of the pelvic floor than rigid laparoscopic instruments permit [42, 43]. The systematic functional advantage of robotic surgery constitutes a quality-of-care argument that is independent of, and additive to, the oncological one [31].

### The randomized trial evidence: from equivalence to superiority

The randomized trial evidence for robotic rectal surgery has evolved through three distinct phases. The ROLARR trial was the first large multicenter randomized controlled trial (RCT) (*n* = 471) to compare robotic and laparoscopic TME in a Western, multi-surgeon setting; it found no significant difference in its primary endpoint of conversion rate (8.1% vs. 12.2%, *p* = 0.16), with subgroup signals favoring robotics in male patients and low tumors [44]. Strictly speaking, ROLARR did not meet its primary endpoint, and it was not powered to detect differences in oncological outcomes; the long-term follow-up has not demonstrated a clear oncological advantage either [44]. Its principal contribution was therefore to establish that robotic TME was safe and feasible in a non-Asian, multi-surgeon setting, rather than to demonstrate superiority.

The COLRAR trial (*n* = 295) conducted in South Korea, found comparable complete TME rates overall but demonstrated superior CRM negativity in the robotic arm specifically in neoadjuvant-treated patients (100% vs. 93.9%, *p* = 0.031)), a clinically meaningful finding limited by early closure due to slow accrual [45].

#### The REAL trial provided the pivotal

advance. The short-term results confirmed non-inferiority and lower conversion rates [46]. The long-term outcomes, published in JAMA in 2025 provided the definitive oncological advance [25]. Conducted at 11 experienced Chinese centers, it enrolled 1,171 patients with middle (> 5–10 cm) or low (0–5 cm) rectal adenocarcinoma (cT1-T3, N0-N1), without distant metastasis, randomized 1:1 to robotic or laparoscopic TME, with surgeons required to have completed their learning curve prior to enrolment. With a median follow-up of 43.0 months, the 3-year locoregional recurrence rate was 1.6% in the robotic group versus 4.0% in the laparoscopic group (HR 0.45, 95% CI 0.22–0.92, *p* = 0.03). Disease-free survival was 87.2% versus 83.4% (HR 0.74, *p* = 0.04). Overall survival was 94.7% versus 93.0% (*p* = 0.16), favoring robotics but not reaching statistical significance [25]. The trial also provided the most comprehensive prospective functional outcome data in a rectal cancer surgery RCT to date: robotic surgery was associated with significantly better urinary function at 3, 6, and 12 months (IPSS, *p* < 0.001), superior sexual function in both males (IIEF-5) and females (FSFI), and improved early defecation control (Wexner scores, *p* < 0.01). Pathological assessment was performed by central pathologists blinded to the surgical arm, mitigating one of the principal sources of bias inherent to non-blindable surgical trials (24).

REAL is a landmark trial [25, 46], because it is the first RCT powered and designed to detect differences in locoregional recurrence, and it found them. Its significance lies in confirming, with prospective randomized evidence, an oncological superiority that the preceding body of meta-analytic and observational evidence had been suggesting. It is the apex of the evidential pyramid, not its foundation (Table 3).

**Table 3:**
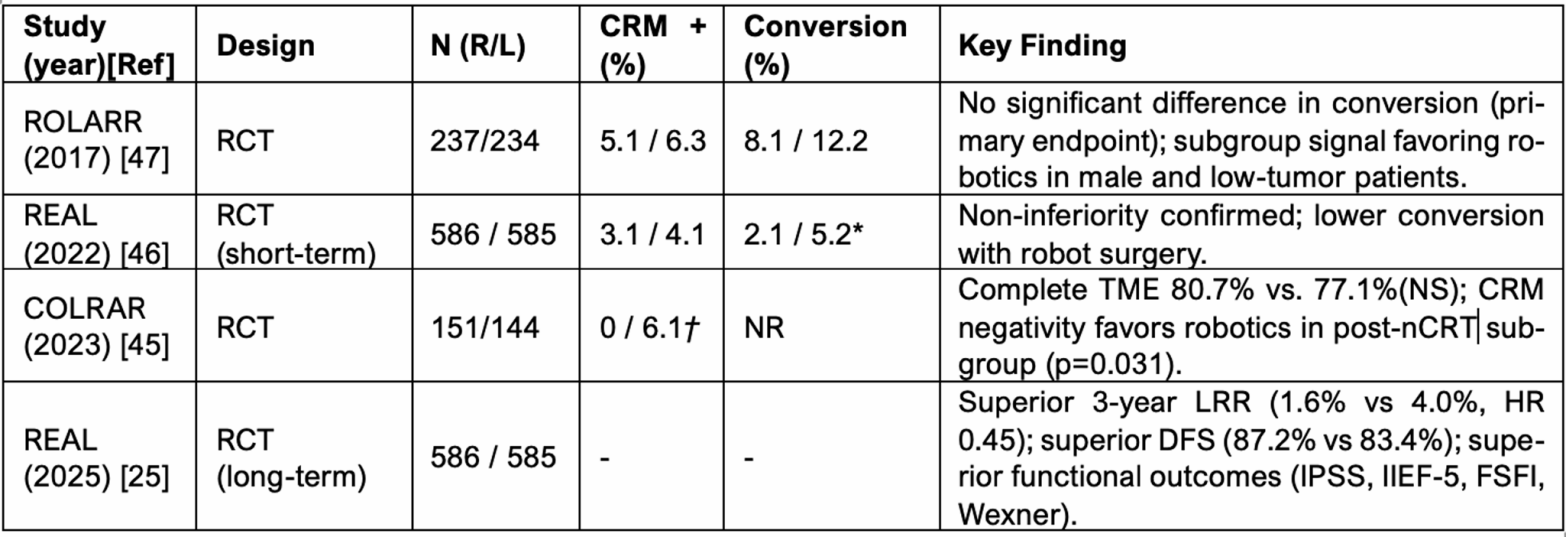
Randomized controlled trials of robotic versus laparoscopic TME for rectal cancer

*R* = robotic; *L* = laparoscopic; *CRM* = circumferential resection margin; *DFS* = disease-free survival; *LRR* = locoregional recurrence rate; *RCT* = randomized controlled trial; *NR* = not reported in primary endpoint. *p = 0.002. †Subanalysis; *CRM* negativity 100% (R) vs. 93.9% (L), *p* = 0.031 in the post-neoadjuvant subgroup

### Cost-effectiveness: evidence is shifting

The traditional argument of robotic surgery’s substantially higher costs without proportional clinical benefit, has been significantly challenged [48]. Early comparative studies did document meaningful cost differentials. A retrospective single-center study comparing robotic-assisted and laparoscopic rectal cancer resections reported significantly higher total inpatient costs (median €17,663 vs. €14,089, *p* = 0.018) and surgery-specific costs (median €10,156 vs. €7,468, *p* < 0.0001) with the robotic approach, resulting in a negative inpatient profit margin (median -€3,196 vs. +€232, *p* = 0.004), despite fewer severe postoperative complications [49]. Similarly, a retrospective comparison of 240 robotic and 258 laparoscopic colorectal cancer resections demonstrated higher overall direct costs ($8,756 vs. $7,776, *p* = 0.001) and supply costs ($3,789 vs. $2,122, *p* < 0.001) with the robotic approach, driven primarily by instrumentation expenses, despite a marginally shorter postoperative length of stay (5.08 vs. 5.55, *p* = 0.113) [50].

However, analyses incorporating broader economic outcomes present a more nuanced picture. A single-surgeon retrospective cohort of 320 consecutive robotic colorectal resections over nine years demonstrated low conversion (1.9%), anastomotic leak (2.2%), and 30-day mortality (1%) rates, with R0 resection in 99.7% of cases. Model-based cost analysis (QTI tool) estimated savings of €483 versus laparoscopy and €3,181 versus open surgery, suggesting that a standardized robotic program may achieve economic sustainability at high volume [48]. A Finnish institutional study further demonstrated that increasing robotic adoption does not necessarily increase total hospital costs when pathway-level savings, from reduced complications, fewer conversions, and shorter rehabilitation, are incorporated into the analysis [29].

The most robust prospective evidence comes from the ROBOCOSTES trial, a national Spanish study specifically designed to evaluate the cost-effectiveness of robotic versus laparoscopic rectal resection. Despite an incremental cost of approximately €920 per case, robotic surgery yielded significantly better quality-adjusted life years (QALYs); the authors concluded that, in the Spanish healthcare context, robotic rectal resection was cost-effective compared with laparoscopy and could be preferred where available [28]. Robotic surgery costs vary substantially between countries and healthcare systems and include not only platform acquisition but also maintenance contracts, service agreements, and per-case consumables. Second-generation platforms (Versius, Hugo, Avatera) are entering clinical use; whether platform competition will translate into meaningful per-case cost reductions remains to be determined [30]; early comparative data with the Hugo RAS platform have already demonstrated clinically equivalent functional and oncological outcomes versus laparoscopy [31].

## The case for robotic TME as the preferred approach in expert centers

### A convergent, multidimensional evidence base

The argument for designating robotic TME as the preferred approach for mid-low rectal cancer in expert centers does not rest on the REAL trial alone. It should be acknowledged at the outset that the comparison underlying this evidence is not between platforms in the abstract but between expert surgeons using each platform; the surgeon factor is a major determinant of outcomes that cannot be fully disentangled from the platform comparison itself. With this caveat in mind, the case is built on convergent evidence across multiple domains: lower conversion rates confirmed across 15 RCTs and tens of thousands of patients in registry studies; superior CRM negativity and TME quality in post-neoadjuvant cases; consistent functional preservation advantages replicated across meta-analyses and now the largest functional outcome dataset in a rectal cancer RCT; emerging survival advantages in observational cohorts; and a cost-utility profile that appears favorable in selected health-system contexts [48, 51, 52].

The REAL trial adds something that the preceding evidence could not provide: a statistically powered demonstration of superior locoregional control in a randomized comparison. In absolute terms, the 3-year locoregional recurrence rate was 1.6% in the robotic group versus 4.0% in the laparoscopic group, an absolute risk reduction of 2.4% (number needed to treat ≈ 42; HR 0.45). The absolute benefit is therefore modest in this favorable-risk population, although the relative reduction is biologically plausible: the robotic platform’s precision in the narrow pelvis facilitates more complete mesorectal excision, lower CRM positivity, and superior autonomic nerve preservation, all of which reduce the anatomical substrate for local recurrence [25]. But this finding confirms a hypothesis already supported by the body of evidence; it does not create the argument *ex nihilo*.

### Technical advantages in anatomically challenging cases

The argument for robotic surgery as the preferred minimally invasive approach is strongest for the patient populations in whom technical difficulty is greatest [53]. Mid-low rectal cancer presents the most demanding anatomical scenario in colorectal surgery: the depth of the pelvis, proximity to the autonomic nerve plexus, sphincter complex, and urogenital structures, and the need for precision in both the posterior mesorectal plane and the anterior dissection adjacent to the prostate or vagina [54].

**Fig. 2:**
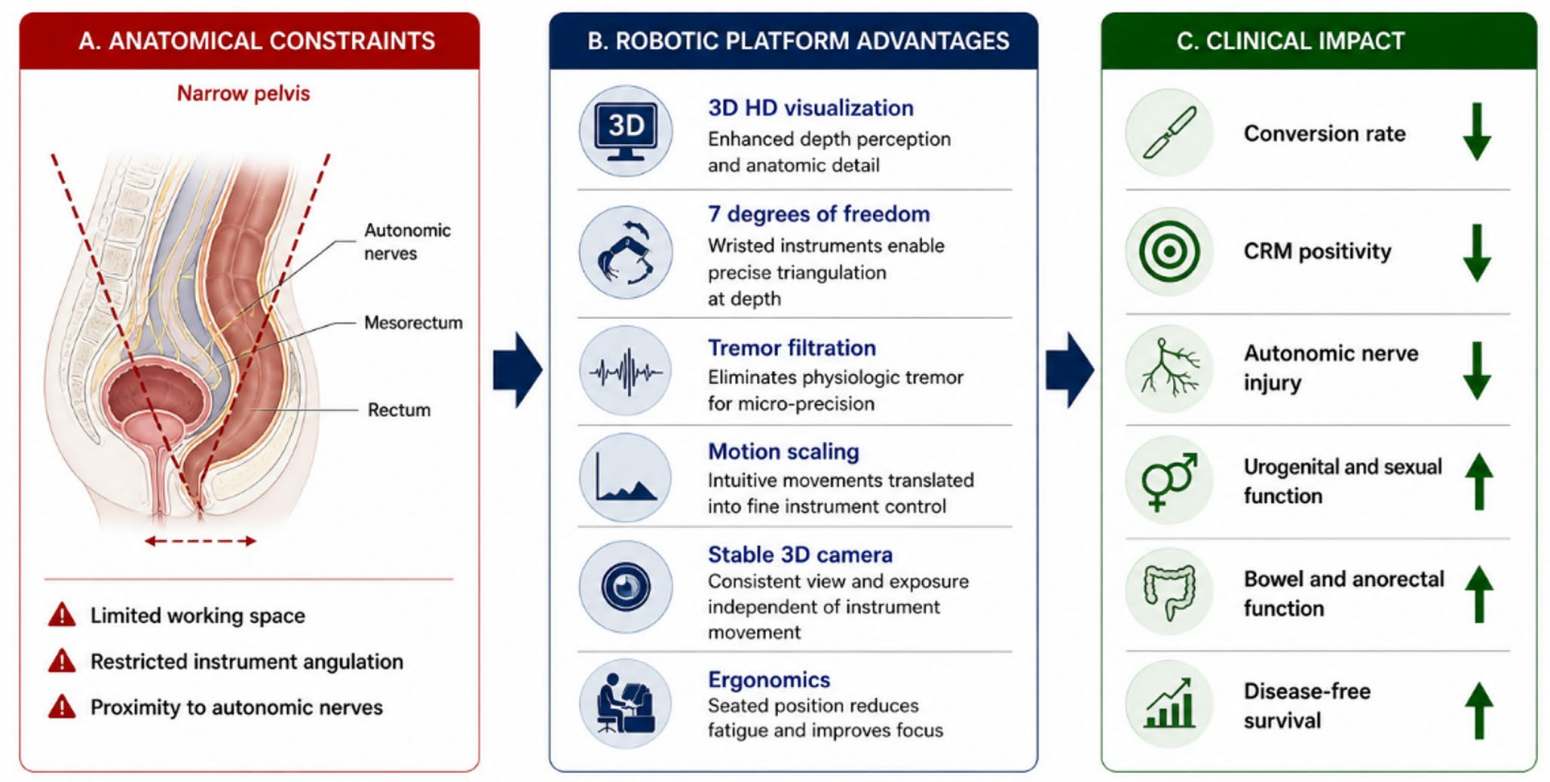
Biomechanical advantages of R-TME in the narrow pelvis and their impact on clinical outcomes. Conceptual framework illustrating how the anatomical constraints of the narrow pelvis impact conventional laparoscopy and how robotic-assisted total mesorectal excision (TME) overcomes these limitations. Restricted working space, limited instrument articulation, and proximity to autonomic nerves increase technical difficulty during laparoscopic dissection. As stated, robotic platforms address these constraints through enhanced visualization, increased degrees of freedom, tremor filtration, and improved ergonomics. These technical advantages translate into improved surgical precision and are associated with lower conversion rates, improved circumferential resection margin (CRM) outcomes, better functional preservation, and improved oncological endpoints in selected patients. *CRM* = circumferential resection margin; *CRT *= chemoradiotherapy; *DFS *= disease-free survival; *DoF* = degrees of freedom; *FSFI *= Female Sexual Function Index; *HR* = hazard ratio; *ICG* = indocyanine green; *IIEF-5* = International Index of Erectile Function-5; *IPSS *= International Prostate Symptom Score; *LRR* = locoregional recurrence; *TME *= total mesorectal excision; *TNT* = total neoadjuvant therapy

The practical consequence is that robotic surgery eliminates or significantly reduces the performance limitations of laparoscopy regarding challenging cases. Conversion to open surgery, with its associated morbidity and potential oncological consequences, is consistently halved. In the most challenging subgroups (male patients, obese patients, post-neoadjuvant tissue fibrosis and low tumors) the advantage is even more amplified [36]. The RESET trial, a prospective European multicenter observational study comparing all four TME approaches (open, laparoscopic, robotic, and TaTME) in 1,078 high-risk patients, found no significant differences between minimally invasive techniques in composite oncological outcomes (CRM $\:\ge\:$ 1 mm, TME grade II-III, and absence of Clavien-Dindo grade III-IV complications) on propensity score analysis; notable, R-TME demonstrated consistent results regardless of center volume, an advantage not observed with laparoscopic or transanal approaches, while including patients with more advanced and more distal tumors, suggesting that robotic surgery absorbed the highest-complexity cases without compromising outcomes [55] (Fig. 2).

### Functional preservation as a quality-of-care imperative

The systematic functional advantages demonstrated by robotic surgery, particularly in the critical 3–12 months postoperative window when neural recovery occurs, represent a quality-of-care imperative beyond the oncological argument. A surgical approach that demonstrably preserves autonomic function better than its alternative, in a population for whom this matters profoundly, has a strong ethical argument for adoption when oncological outcomes are at least equivalent, and an even stronger argument when they are superior.

## Remaining challenges and counterarguments

### The generalizability question: can the REAL trial travel?

The most substantive challenge to extrapolating the REAL trial’s findings to a global standard-of-care recommendation is the question of generalizability. The trial was conducted exclusively at Chinese high-volume centers by surgeons who had completed their robotic learning curve. The operative setting, experienced surgeons, high caseload, structured quality assurance of TME technique, may not reflect practice conditions in lower-volume centers, early-adoption hospitals, or health systems where robotic is being introduced for the first time.

This concern is mitigated, but not eliminated, by the broader evidence base. The conversion rate advantage of robotic surgery is observed across Western multicenter trials (ROLARR) and diverse registry cohorts (EUREKA), suggesting it is not confined to Chinese high-volume settings. Even so, the oncological superiority demonstrated in the REAL trial may be primarily reproducible in centers that have moved beyond the learning curve. This is a powerful argument for structured training programs, proctorship, and volume thresholds as prerequisites, not an argument against robotic adoption, but a clear argument for how it should be implemented. Indeed, a recent narrative overview from leading European colorectal robotic surgeons reaffirms that, despite two decades of clinical implementation, the strongest evidence for robotic superiority emerges precisely when surgeon-experience bias is controlled, as in REAL trial, and emphasizes that ergonomic and connectivity advantages for surgeons should be incorporated into the broader benefit-risk calculus alongside patient-level outcomes [56].

### Learning curve: a non-trivial barrier

The learning curve for robotic rectal surgery is substantial, with published estimates ranging from 20 to 50 cases before performance metrics plateau [57–59]. Unlike laparoscopy, which is now established in surgical training programs worldwide, robotic training requires access to simulators, dedicated proctorship, and institutional investment in protected training time. The REAL trial is silent on learning curve effects, having required surgeons to have completed their learning curve prior to enrolment. Real-world implementation must navigate this transitional phase carefully [25, 46].

More recent structured analyses, incorporating multidimensional performance metrics and control chart methodology, suggest that the learning curve for robotic TME may be somewhat shorter than for advanced laparoscopic TME, and importantly, that the performance plateau is higher, reflecting the ergonomic advantages of the robotic platform once proficiency is achieved [60].

A systematic review examining learning curves for robotic colorectal surgery found that the learning curve for robotic TME ranged from 15 to 40 cases across studies, with considerable variation depending on the surgeon’s metrics used to define proficiency [61]. Surgeons with advanced laparoscopic colorectal experience appear to acquire robotic competency more rapidly than those transitioning from open surgery, suggesting that foundational minimally invasive skills facilitate platform specific adaptation.

Structured training curricula, simulation-based competency assessment, and mandatory proctorship for new robotic rectal surgeons should be considered minimum requirements before independent practice [62–64]. Simulation platforms, including virtual reality robotic surgical simulators and cadaveric or animal model curricula, provide trainees with opportunities prior to clinical exposure [65]. The recently published ColoRobotica framework provides a structured, modular curriculum for robotic colorectal surgery that may serve as a template for European-wide standardization [63]. Within these training frameworks, video-based proficiency-based progression (PBP) binary metrics have shown superior inter-rater reliability and stronger discrimination between novice and experienced surgeons compared with global rating scales such as GEARS (Global Evaluative Assessment of Robotic Skills), particularly during the critical pelvic dissection phase, supporting their incorporation into objective competency assessment for robotic colorectal training [66]. The evidence supports robotic surgery as standard of care for expert practitioners; it does not support it as an approach that can be adopted without appropriate preparation.

### Robotic-specific considerations and complications

Adoption of any surgical platform requires explicit consideration of its specific complication profile. Robotic platforms are complex systems susceptible to technical failure, with reported malfunction rates ranging from 0.4% to 4.6%. The most frequently described failures include setup joint dysfunction, robotic arm malfunction, electrical arcing, and instrument fragmentation with retention in the surgical field. The absence of haptic feedback represents an inherent limitation, potentially leading to inadvertent tissue damage through uncontrolled force application. Optimal equipment maintenance, prompt failure reporting, and standardized team training are essential to minimize technical complications and avoid unnecessary conversion or procedure cancellation [67]. Robotic rectal surgery is associated with longer total operative times in some series, particularly during the early learning curve, with potential implications for elderly or frail patients in whom prolonged anesthesia carries additional risk [60, 61]. Port-site complications, including hernia and trocar-site bleeding, are reported at rates broadly comparable to laparoscopy, although the larger 8-mm and 12-mm robotic ports may warrant routine fascial closure. Reliance on the docked platform introduces specific intraoperative considerations: undocking for emergent conversion is generally rapid in modern systems but remains a procedural step absent in laparoscopy. Surgeon ergonomics, frequently cited as a robotic advantage, may reduce musculoskeletal strain over a career, although this benefit has been formally quantified in only a limited number of studies [43]. None of these considerations alters the overall risk-benefit balance described above, but they should be incorporated into informed consent and institutional preparedness.

### The absence of overall survival benefit

The REAL trial did not demonstrate a statistically significant improvement in overall survival (OS) (94.7% vs. 93.0%, *p* = 0.16) [25]. This finding warrants careful interpretation. The trial was powered to detect differences in locoregional recurrence, not overall survival; the absence of statistically significant OS benefit should not be interpreted as evidence of equivalence. Overall survival at 3 years in a T1-T3, N0-N1 rectal cancer population treated with modern multimodal therapy already exceeds 93%, leaving limited statistical room to detect differences. At 3 years, most OS events result from distant metastases, not locoregional recurrence, and the follow-up is simply insufficient to judge OS impact. Five-year data will be essential to determine whether the locoregional advantage translates into a survival benefit.

### Cost, access, and global equity

Even accepting the cost-effectiveness argument, the capital investment required for robotic platforms, €1–2 million for acquisition, plus substantial per-case running costs, creates an access barrier that cannot be dismissed. In healthcare systems with constrained capital budgets, the opportunity cost of robotic platform investment must be considered against competing priorities. In low- and middle-income countries, where rectal cancer incidence is rising most rapidly due to westernization of lifestyle, robotic surgery remains practically inaccessible for the foreseeable future.

A global standard-of-care recommendation that implicitly requires robotic infrastructure risks creating a two-tiered standard of care, one for well-resourced health systems and one for the remainder of the world. The surgical community’s response to this challenge should be to advocate simultaneously for the preferred status of robotic surgery in systems where it is accessible, and for the continued excellence of laparoscopic TME, which remains an oncologically sound approach, in settings where robotic adoption is not feasible. The goal should be progressive convergence towards robotic surgery as resources permit, not premature declaration of a standard that excludes the majority of the world’s patients.

### TaTME: complementary rather than competing

Transanal TME (TaTME) has emerged as an alternative minimally invasive approach that specifically addresses the anatomical limitations of abdominal laparoscopy in the low rectum, approaching pelvic dissection from below. Comparative evidence between robotic TME and TaTME remains limited, a meta-analysis of five prospective studies (*n* = 1,941) found no significant differences in operative duration, hospital stay, or major complications [68]. The first prospective pilot series of robotic TaTME (R-TaTME), reported in 2015, demonstrated technical feasibility with complete mesorectal excision and negative margins in all five patients, establishing the anatomical and technical premise of the approach [69]. R-TaTME has subsequently been proposed as a hybrid technique combining the oncological rationale of perineal dissection with the precision and dexterity of the robotic platform, potentially mitigating the anastomotic leak and local recurrence concerns raised in national TaTME series; however, long-term oncological and functional outcome data remain insufficient to define its role [70]. TaTME may offer specific advantages for ultra-low tumors where transabdominal access is most constrained, and the two approaches may ultimately serve complementary rather than competing roles. The standard-of-care question for rectal cancer surgery should not be framed as robotic versus TaTME but rather as the appropriate allocation of each approach based on tumor location, patient anatomy, and institutional expertise.

## Who should be operated robotically in 2026? A practical framework

Based on the evidence reviewed, we propose the following framework for surgical approach selection in rectal cancer in 2026, acknowledging that it must be contextualized by institutional expertise, resource availability, and patient-specific factors (Table 4).

**Table 4:**
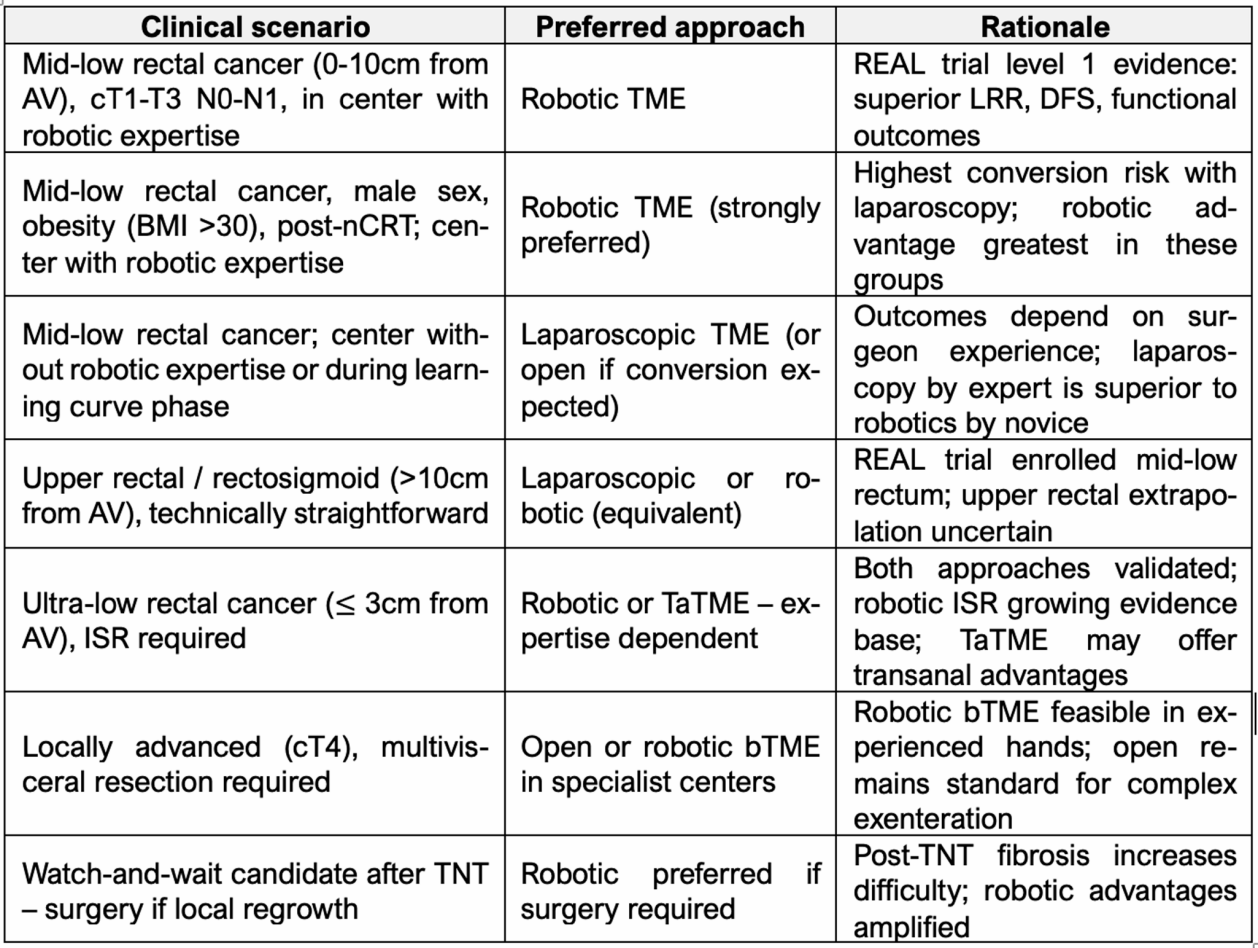
Proposed surgical approach framework for rectal cancer in 2026

*AV* = anal verge; *LRR* = locoregional recurrence rate; *DFS* = disease-free survival; *nCRT* = neoadjuvant chemoradiotherapy; *ISR* = intersphincteric resection; *TME* = total mesorectal excision; *bTME* = beyond total mesorectal excision; *TaTME* = transanal total mesorectal excision; *TNT* = total neoadjuvant therapy

Central to this framework is the principle that surgical approach selection must be guided by institutional and individual competence, not platform availability alone. The designation of robotic TME as the preferred approach in expert centers does not imply that robotic surgery in inexperienced hands is preferable to expert laparoscopy. Credentialling, proctorship, and volume-outcome standards are prerequisites for safe implementation as the preferred approach (Table 5).

**Table 5:**
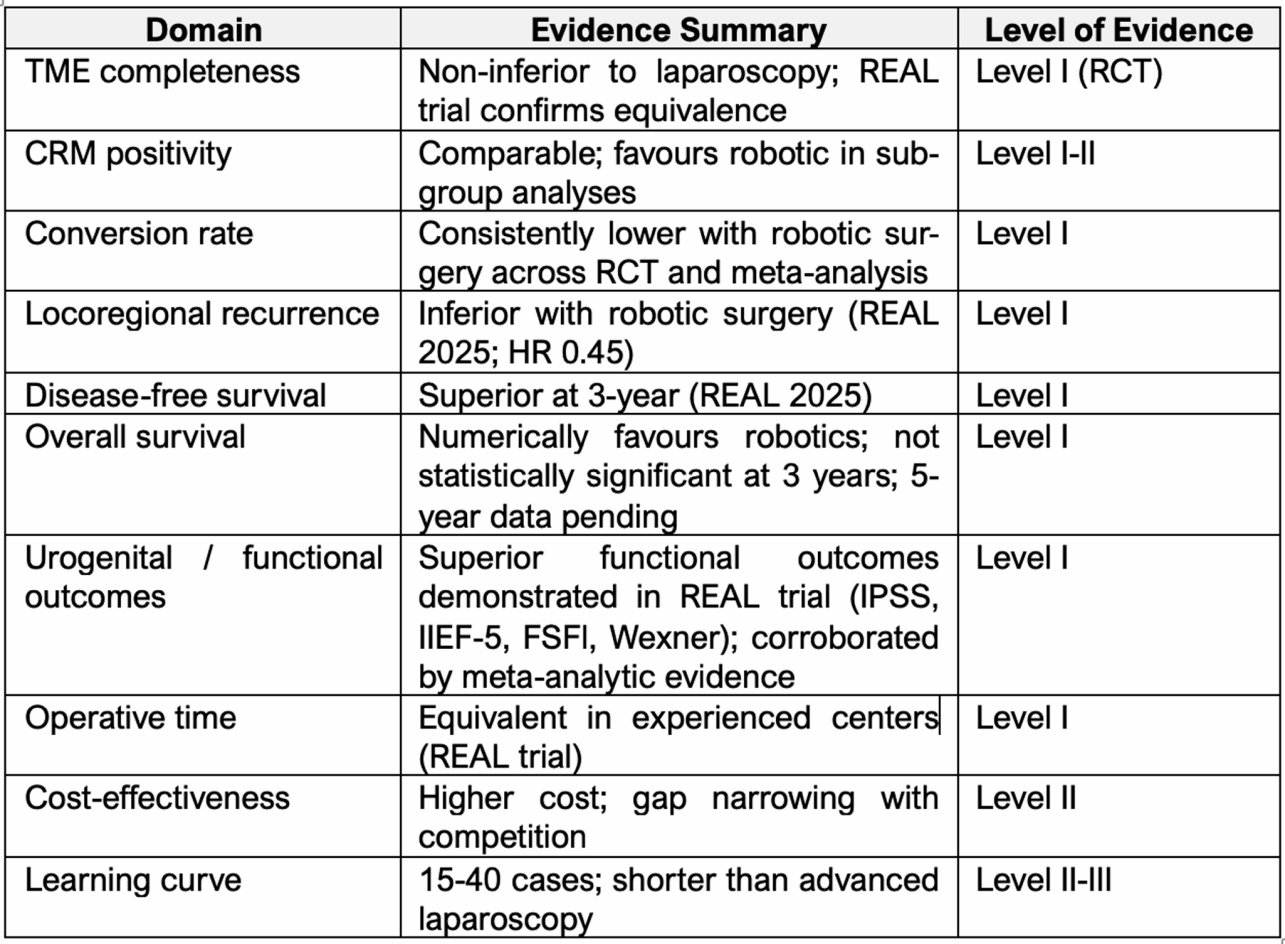
Summary of evidence: robotic vs. laparoscopic TME for rectal cancer in 2026

*TME* = total mesorectal excision; *CRM* = circumferential resection margin; *DFS* = disease-free survival; *OS* = overall survival; *RCT *= randomized controlled trial

## Future directions

Several critical questions remain to be answered as the field evolves. First, 5-year follow-up data from the REAL trial are required to determine whether the locoregional advantage translates into an overall survival benefit, the most clinically definitive endpoint. Second, replication of the REAL trial findings in Western healthcare contexts, with diverse surgeon experience levels, different health systems, and different patient demographics, is essential before global standard-of-care designation can be made with confidence. An European or North American analogue of the REAL trial, adequately powered and with standardised surgeon credentialling, should be considered a research priority.

Third, head-to-head comparisons between robotic TME and TaTME in defined patient subgroups, particularly ultra-low rectal cancer and post-neoadjuvant resections, are needed to refine the indication boundaries of each approach. Fourth, the integration of robotic surgery with emerging perioperative strategies, including total neoadjuvant therapy and organ preservation protocols, requires prospective evaluation, as the watch-and-wait landscape changes the context in which surgery is performed and the technical requirements of salvage resection. Fifth, comparative effectiveness data with second-generation robotic platforms (Hugo RAS, Versius, Avatera) are urgently needed: virtually all current high-quality evidence comes from da Vinci-based studies, and the generalisability for REAL-trial findings across platforms cannot be assumed.

Finally, the development of standardised robotic training curricula, simulation-based competency assessment tools, and certification frameworks will be essential to ensure that the outcomes demonstrated in the REAL trial are reproducible in the broader surgical community. The standard of care cannot be defined only by what is achievable in expert centers; it must be achieved, with appropriate training, across a spectrum of centers treating rectal cancer.

## Conclusions

The question posed in the title of this review “Is robotic surgery the standard of care for rectal cancer in 2026?” admits no single, context-free answer. The evidence supports a conditional and qualified affirmation.

In centers with established robotic expertise, adequate case volume, completed surgeon learning curves, and structured quality assurance of TME technique, robotic surgery may reasonably be considered the preferred approach for mid-low rectal cancer. The REAL trial has provided level 1 evidence of superior locoregional control, improved disease-free survival, and better functional outcomes with robotic compared to laparoscopic TME. These findings, integrated with consistent meta-analytic evidence of lower conversion rates, comparable to favorable TME quality, and advantages in pelvic nerve preservation, provide a coherent and multidimensional case for robotic TME as the preferred surgical approach in this setting.

In centers without robotic expertise, health systems where robotic surgery is not accessible, and in early-adoption programmes where learning-curve effects have not yet been overcome, expert laparoscopic TME, performed by experienced surgeons in high-volume centers, remains an oncologically sound and clinically appropriate approach. The transition to robotic surgery, when undertaken, should be structured, proctored, and credentialled rather than opportunistic.

Several caveats temper the strength of any global recommendation. The pivotal REAL trial was conducted exclusively at Chinese high-volume centers, and replication in Western settings remains a research priority. Three-year overall survival did not reach statistical significance, and 5-year data will be needed to confirm whether the locoregional advantage translate into a survival benefit. Cost and access barriers continue to limit equitable adoption, particularly in low- and middle-income healthcare systems.

Taken together, the evidence in 2026 supports robotic TME as the preferred minimally invasive approach for mid-low rectal cancer when the conditions of expertise, infrastructure, and case volume are met. Where these conditions are not met, expert laparoscopy retains its place. A standard-of-care recommendation that depends on a single trial is structurally fragile; one grounded in convergent evidence from multiple sources and methodologies, while explicitly acknowledging its remaining uncertainties, offers a more durable basis for clinical practice.

## Data Availability

No datasets were generated or analysed during the current study.
